# Can computed tomography-based radiomics potentially discriminate between anterior mediastinal cysts and type B1 and B2 thymomas?

**DOI:** 10.1186/s12938-020-00833-9

**Published:** 2020-11-27

**Authors:** Lulu Liu, Fangxiao Lu, Peipei Pang, Guoliang Shao

**Affiliations:** 1 Institute of Cancer and Basic Medicine (ICBM), Chinese Academy of Sciences, Hangzhou, China; 2 Department of Radiology, Cancer Hospital of the University of Chinese Academy of Sciences, Hangzhou, China; 3 Department of Radiology, Zhejiang Cancer Hospital, No. 1 Banshan Street, Gongshu District, Hangzhou, 321022 Zhejiang People’s Republic of China; 4 Life Sciences, GE Healthcare, Hangzhou, 310000 Zhejiang China

**Keywords:** Anterior mediastinal cysts, Thymomas, Radiomics, Enhanced CT

## Abstract

**Background:**

Anterior mediastinal cysts (AMC) are often misdiagnosed as thymomas and undergo surgical resection, which caused unnecessary treatment and medical resource waste. The purpose of this study is to explore potential possibility of computed tomography (CT)-based radiomics for the diagnosis of AMC and type B1 and B2 thymomas.

**Methods:**

A group of 188 patients with pathologically confirmed AMC (106 cases misdiagnosed as thymomas in CT) and thymomas (82 cases) and underwent routine chest CT from January 2010 to December 2018 were retrospectively analyzed. The lesions were manually delineated using ITK-SNAP software, and radiomics features were performed using the artificial intelligence kit (AK) software. A total of 180 tumour texture features were extracted from enhanced CT and unenhanced CT, respectively. The general test, correlation analysis, and LASSO were used to features selection and then the radiomics signature (radscore) was obtained. The combined model including radscore and independent clinical factors was developed. The model performances were evaluated on discrimination, calibration curve.

**Results:**

Two radscore models were constructed from the unenhanced and enhanced phases based on the selected four and three features, respectively. The AUC, sensitivity, and specificity of the enhanced radscore model were 0.928, 89.3%, and 83.8% in the training dataset and 0.899, 84.6%, and 87.5% in the test dataset (higher than the unenhanced radscore model). The combined model of enhanced CT including radiomics features and independent clinical factors yielded an AUC, sensitivity and specificity of 0.941, 82.1%, and 94.6% in the training dataset and 0.938, 92.3%, and 87.5% in the test dataset (higher than the unenhanced combined model and enhanced radscore model).

**Conclusions:**

The study suggested the possibility that the combined model in enhanced CT provided a potential tool to facilitate the differential diagnosis of AMC and type B1 and B2 thymomas.

## Background

Thymoma is the most common tumour in the anterior mediastinum [1, 2]. According to the 2015 World Health Organization (WHO) classification of thymic epithelial tumours, thymomas are no longer classified as benign tumours. Except for micronodular thymoma with lymphoid stroma and micro-thymomas are benign, all other types of thymoma are considered malignant tumours [3]. Thus, when the mediastinal mass is suspected to be a thymoma, surgical resection is needed [4, 5].

Currently, routine computed tomography (CT) is widely used as a routine method diagnosing lesions in the thymoma and anterior mediastinal cysts (AMC). Type A and AB thymomas have many thymic epithelial cells and are heterogeneous on enhanced CT imaging. Type B3 thymomas and thymic carcinomas may invade the surrounding structures due to their high invasiveness, and most of them can easily be distinguished from cysts preoperatively. Otherwise, type B1 and B2 thymomas comprise many lymphocyte cells and even enhancement on enhanced CT imaging, and thymoma often have a complete capsule, making it difficult to distinguish them from AMC [6–8]. Meanwhile, the CT value of some AMC may be similar to the soft tissue density, due to the influence of mediastinal large blood vessels and the thorax, the CT values of the enhanced mediastinal window are not accurate, and some are even lower than the unenhanced CT values [9, 10], which make the diagnoses mainly based on radiologists’ subjective experience. Thus, patients with AMC are often misdiagnosed as thymomas and undergo surgical resection, not only causing unnecessary treatment but also wasting medical resources. In this study, 106 included cases of AMC were all misdiagnosed as thymomas and underwent surgical resection.

Radiomics can extract a high-throughput objective and quantitative image features from CT, magnetic resonance imaging (MRI), or positron emission tomography (PET) to reflect tumour heterogeneity [11–13] and explore the potential relationships between features and pathophysiology to predict clinical outcomes, such as differential diagnosis, classification, distant metastases, survival [14–17]. A few studies discussed the role of quantitative image analysis based on magnetic resonance imaging (MRI) parameters in differentiation anterior mediastinal cysts from other solid masses, which may help to characterize correlation of thymic epithelial tumours with World Health Organization classification and clinical staging [18, 19]; Qualitative CT radiomics analysis were also applied to thymic tumours grading [20]; however, radiomics-based studies of only AMC distinguished from type B1 and B2 thymomas have not been reported. Therefore, this retrospective study attempts to study whether CT radiomics can reflect the heterogeneity between AMC and type B1 and B2 thymoma and to avoid the resection of AMC that are misdiagnosed as thymomas.

## Results

### Patient characteristics

**Fig. 1:**
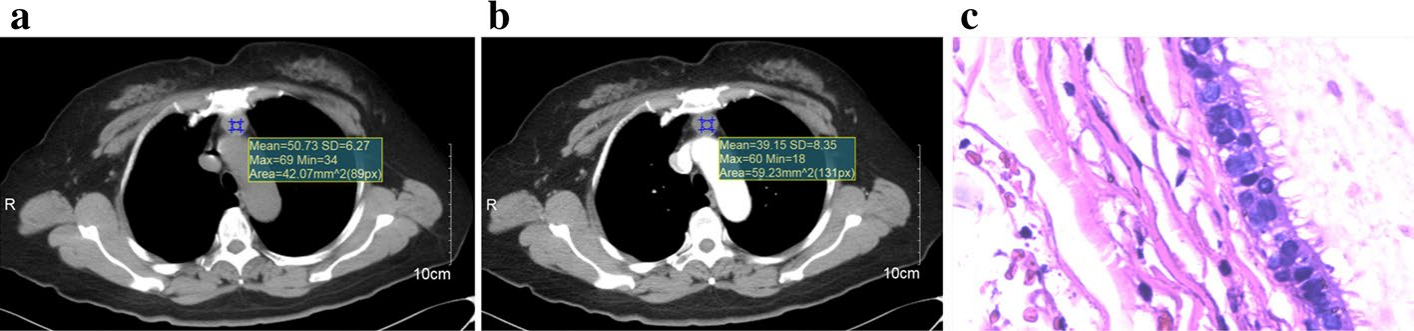
A patient pathologically confirmed anterior mediastinal cyst. **a** Round lesion located in the anterior mediastinum; **b** CT value of 50.73 HU in the unenhanced mediastinal window, CT value of 39.15 HU in the enhanced mediastinal window, even lower than the unenhanced CT value; **c** light microscopy of frozen section of a lesion stained, original magnification × 100 showing the ciliated columnar epithelium lining the lesion capsule

106 anterior mediastinal cysts patients (all were misdiagnosed as thymomas in CT) and 82 type B1 and B2 thymomas patients were included in the study. A patient pathologically confirmed AMC but was misdiagnosed as thymoma in CT image was shown in Fig. 1.

Detail clinical features in training and test dataset were described in Table 1. The maximum diameter [21] of the tumour was 0.9–7.7 cm, and the average diameter was 3.0 ± 1.4 cm. A statistically significant difference was found in lesion size and unenhanced CT value between AMC and thymomas in the training dataset, but no statistically significant difference in age and primary site (*P* > 0.05). There was a significant difference found in enhanced CT value, change of CT value and radscore in the training and test datasets. Moreover, the radscore was the dominant factor impacting the prediction of the differential diagnosis between AMC and type B1 and B2 thymomas (*P* < 0.001).

**Table 1:** Characteristics of patients in the training and test datasets

Characteristics	Training dataset (*N* = 130)			Test dataset (*N* = 58)		
	AMC (*N* = 74)	Thymoma (*N* = 56)	*P*	AMC (*N* = 32)	Thymoma (*N* = 26)	*P*
Age (years)	54.19 ± 8.05	50.71 ± 9.17	0.110	55.81 ± 9.52	44.62 ± 13.04	0.013
Gender			0.213			0.577
Male	34(45.9%)	18(32.1%)		20 (62.5%)	12 (46.2%)	
Female	40 (54.1%)	38 (67.9%)		12(37.5%)	14 (53.8%)	
Primary site			0.301			0.041
Left	44 (59.5%)	30 (53.6%)		22 (68.7%)	18 (69.2%)	
Right	30 (40.5%)	26 (46.4%)		10 (31.3%)	8 (30.8%)	
Lesion size (cm)	2.72 ± 1.40	3.41 ± 1.26	0.043	2.60 ± 1.34	3.52 ± 1.24	0.069
Unenhanced CT value	33.14 ± 17.59	43.34 ± 9.76	0.004	27.90 ± 17.14	38.06 ± 9.21	0.053
Enhanced CT value	35.26 ± 18.19	67.74 ± 16.34	**0.000**	34.40 ± 20.04	57.07 ± 16.61	**0.003**
Change of CT value	2.13 ± 12.45	24.40 ± 12.67	**0.000**	6.50 ± 14.68	19.01 ± 13.25	**0.025**
Radscore	− 2.52 ± 2.22	2.30 ± 2.72	**0.000**	− 2.41 ± 1.77	1.96 ± 2.66	**0.000**

Bold indicates* p* values below 0.05 were considered significant in both training and test datasets

Continuous variables: represented as mean ± standard deviation (SD); Chi-square test or Fisher’s exact test [number and percentage (%)]

*CT* computed tomography, *HU* Hounsfield unit, *change of CT value* enhanced CT value − unenhanced CT value, *AMC* anterior mediastinal cysts

### Radscore model development and performance evaluation

**Fig. 2:**
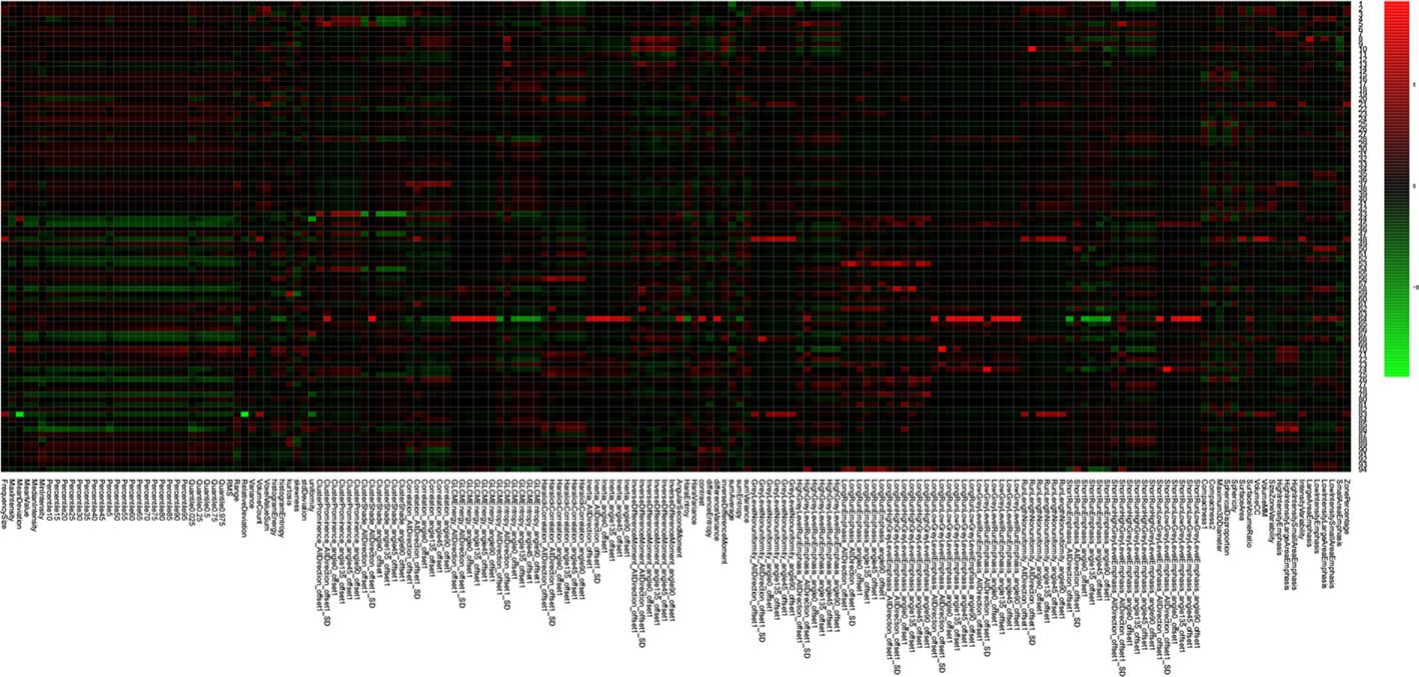
Radiomics heatmap of the extracted 180 texture features. Unsupervised clustering of patients (*n *= 188) on the *y*-axis and expression of radiomics features (*n* = 180) on the *x*-axis reveal clusters of patients with similar radiomic expression patterns. The groups of these radiomics features were labelled on the right side

Among the 180 features of enhanced CT, the correlation based on the heatmap, two main clusters of 188 patients were compared, and a visual association was found, demonstrating the potential discriminative power of these radiomics features (Fig. 2).

**Fig. 3:**
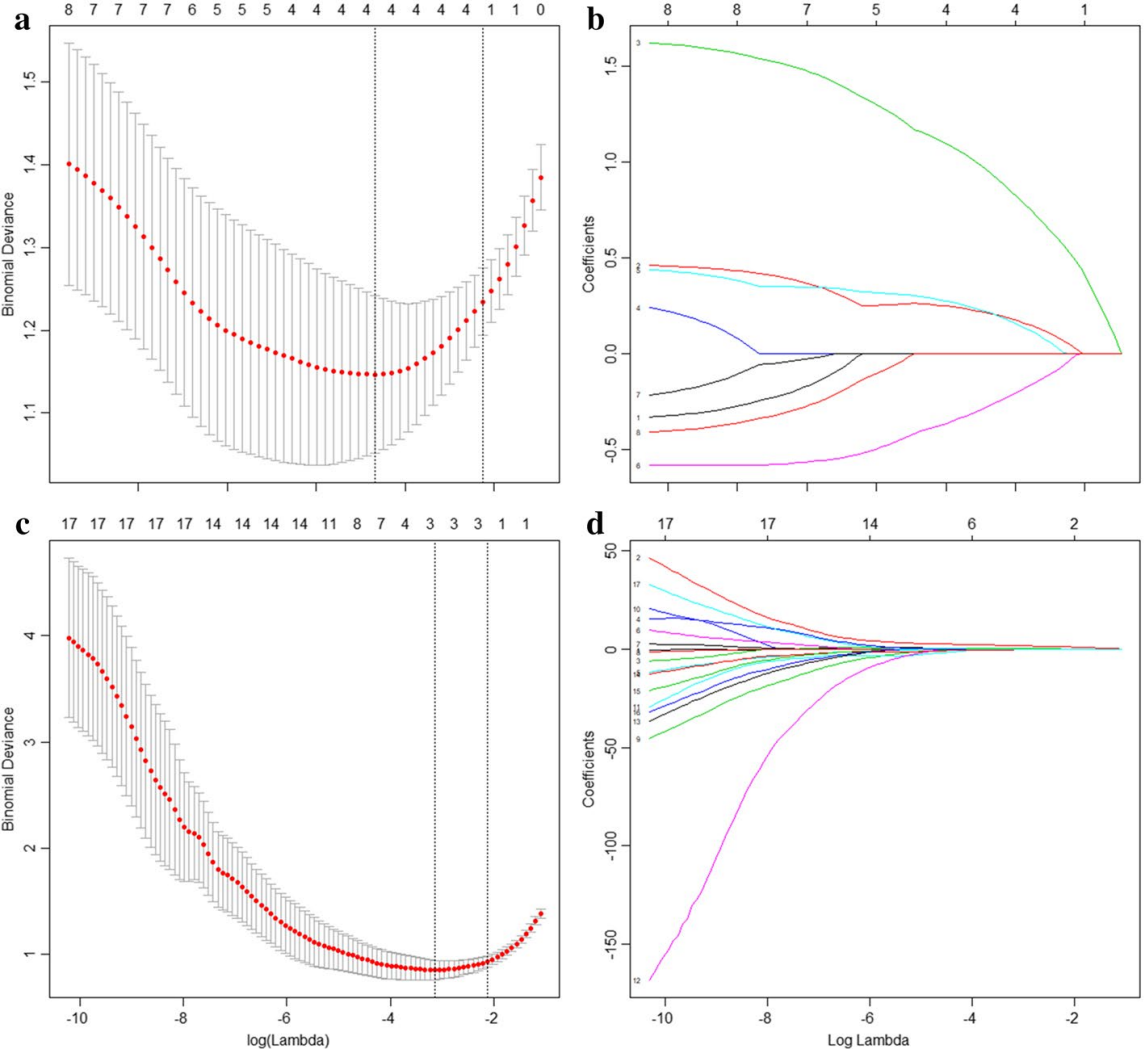
Feature selection using LASSO to shrink some regression coefficients to exactly zero (loss function ${\text{minimize}}\left\{ {\frac{1}{2N}\sum\nolimits_{i = 1}^{N} {(y_{i} - \beta_{0} - \sum\nolimits_{j = 1}^{p} {x_{ij} } \beta_{j} )^{2} + \lambda \left\| \beta \right\|_{{l_{1} }} } } \right\}$. $\lambda$ is used to limit $\sum\nolimits_{j = 1}^{p} {\left| {\beta_{j} } \right| \le t}$). Ten-time cross-validations were used to determine the optimal values of tuning parameter (*λ*). We selected *λ* via 1-SE (standard error). The optimal *λ* is the largest value for which the partial likelihood deviance is within one SE of the smallest value of partial likelihood deviance. **a**, **c** Tuning parameter (*λ*) selection in the LASSO model shown versus log (*λ*). Dotted vertical lines were drawn at the optimal values using the minimum binomial deviation value, log (*λ*) = − 3.38 in unenhanced CT and log (*λ*) = − 3.67 in enhanced CT; **b**, **d** LASSO coefficient profiles of the 180 texture features. A coefficient profile plot was produced against the log (*λ*) sequence. Option l resulted in four nonzero coefficients on unenhanced phase CT imaging and three nonzero coefficients on enhanced phase CT imaging

After the feature selection, four and three imaging features were finally selected from unenhanced phase and enhanced phase CT, respectively, for the construction of the radscore model. The VIFs of four features in unenhanced CT and three features in enhanced phase CT were all less than ten, which indicated no severity collinearity. The lasso images of the unenhanced and enhanced phases were shown in Fig. 3.

Equation 1 represents the unenhanced phase CT for four radiomics features:

1 $$ {\text{radscore}} = 0.{293} \times {\text{Quantile}}0.0{25} + {1}.{3}0{6} \times {\text{VoxelValueSum}} + 0.{356} \times {\text{GLCMEntropy}}\_{\text{angel}}0\_{\text{offset1}} - 0.{532} \times {\text{HaralickCorrelation}}\_{\text{AllDirection}}\_{\text{offset1}}\_{\text{SD}} - 0.{278}. $$

Equation 2 represents the enhanced phase CT for three radiomics features:

2 $$ {\text{Radscore}} = {2}.{7}0{6} \times {\text{RMS}} + 0.{52}0 \times {\text{VoxelValueSum}} - 0.{951} \times {\text{SurfaceVolumeRatio}} - 0.{447}{\text{.}} $$

The AUC, sensitivity, and specificity of the enhanced radscore model were 0.928, 89.3%, and 83.8%, respectively, in the training dataset and 0.899, 84.6%, and 87.5%, respectively, in the test dataset (higher than the unenhanced radscore model) (Table 2). The AUC values were consistent with the results of 1000 times bootstrap analysis in both training and test dataset (mean ± standard deviation: training dataset: 0.928 ± 0.025; test dataset: 0.899 ± 0.039).

**Table 2:** Differential diagnostic efficiency between AMC and type B1 and B2 thymomas

	AUC	95% CI	Sensitivity	Specificity	Youden	*P* value
Radscore model						
Unenhanced CT						
Training dataset	0.823	0.709–0.907	0.757	0.821	0.578	**0.000**
Test dataset	0.856	0.676–0.958	1	0.769	0.769	**0.000**
Enhanced CT						
Training dataset	0.928	0.835–0.977	0.893	0.838	0.731	**0.000**
Test dataset	0.899	0.730–0.979	0.846	0.875	0.721	**0.000**
Combined model						
Unenhanced CT						
Training dataset	0.933	0.843–0.980	0.893	0.838	0.731	**0.000**
Test dataset	0.928	0.768–0.991	0.923	0.875	0.798	**0.000**
Enhanced CT						
Training dataset	0.941	0.853–0.984	0.821	0.946	0.767	**0.000**
Test dataset	0.938	0.781–0.993	0.923	0.875	0.798	**0.000**

Bold indicates* p* values below 0.05 were considered significant in both training and test datasets

### Development of combined model and models comparison

Two clinical unenhanced CT value and enhanced CT value were selected from seven clinical features to develop the clinical models by stepwise multivariable analysis using the minimum Akaike information criterion (AIC) as the stop rule. A combined model including radscore, unenhanced CT value and enhanced CT value was constructed. The VIFs of combined models were calculated, the values all were less than ten, which indicated no severity collinearity.

Equation 3 represents the unenhanced phase CT for combined model:

3 $$ {\text{Combine - model}} = 0.{822} \times {\text{radscore}} + 0.{127} \times {\text{ enhanced CT value}} - 0.0{74} \times {\text{ unenhanced CT value}} - {3}.{443}{\text{.}} $$

Equation 4 represents the enhanced phase CT for combined model:

4 $$ {\text{Combine - model}} = 0.{633} \times {\text{radscore}} + 0.0{83} \times {\text{ enhanced CT value}} - 0.0{53} \times {\text{ unenhanced CT value}} - {2}.{319}{\text{.}} $$

**Fig. 4:**
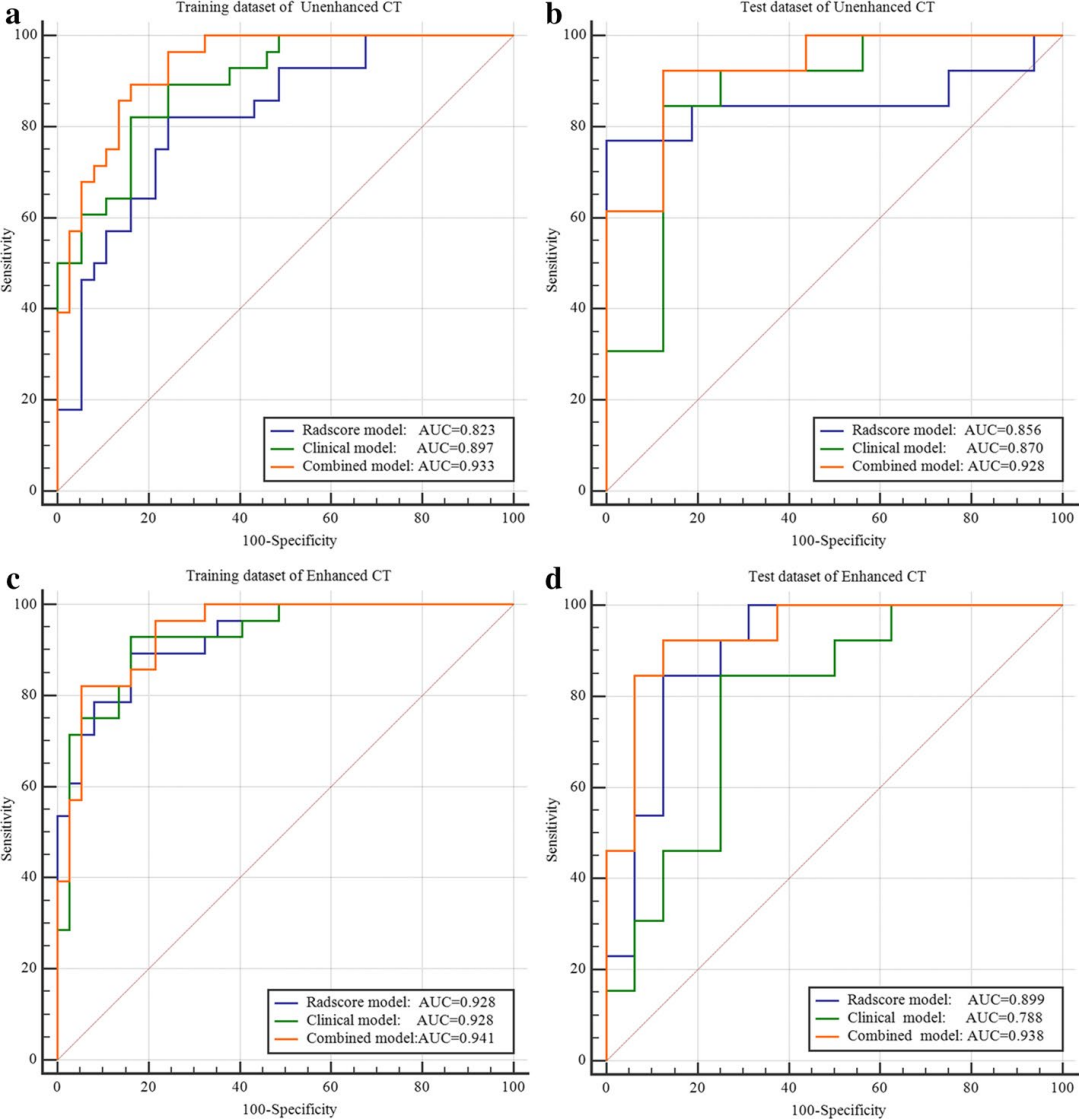
ROC curves of the models. **a**, **b** The clinical model, radscore model and combined model of the unenhanced phase in the training dataset (AUC = 0.897, 0.823, 0.933) and test dataset (AUC = 0.870, 0.856, 0.928); **c**, **d** the clinical model, radscore model and combined model of the enhanced phase in the training dataset (AUC = 0.928, 0.928, 0.941) and test dataset (AUC = 0.788, 0.899, 0.938)

The performance of clinical models, radscore models, and combined models of the unenhanced and enhanced phases CT were evaluated by ROC (Fig. 4). The AUC, sensitivity, specificity, 95% confidence interval (CI), *P* value, Youden index of the training and test datasets of the unenhanced model and enhanced model were detailly shown in Table 2. The AUC of combined model was greater than radscore model and clinical model in two datasets of enhanced and unenhanced CT, the combined model of enhanced CT was the highest. So, the diagnosis efficiency of enhanced model was better than unenhanced model.

### Combined nomogram of the enhanced CT model

**Fig. 5:**
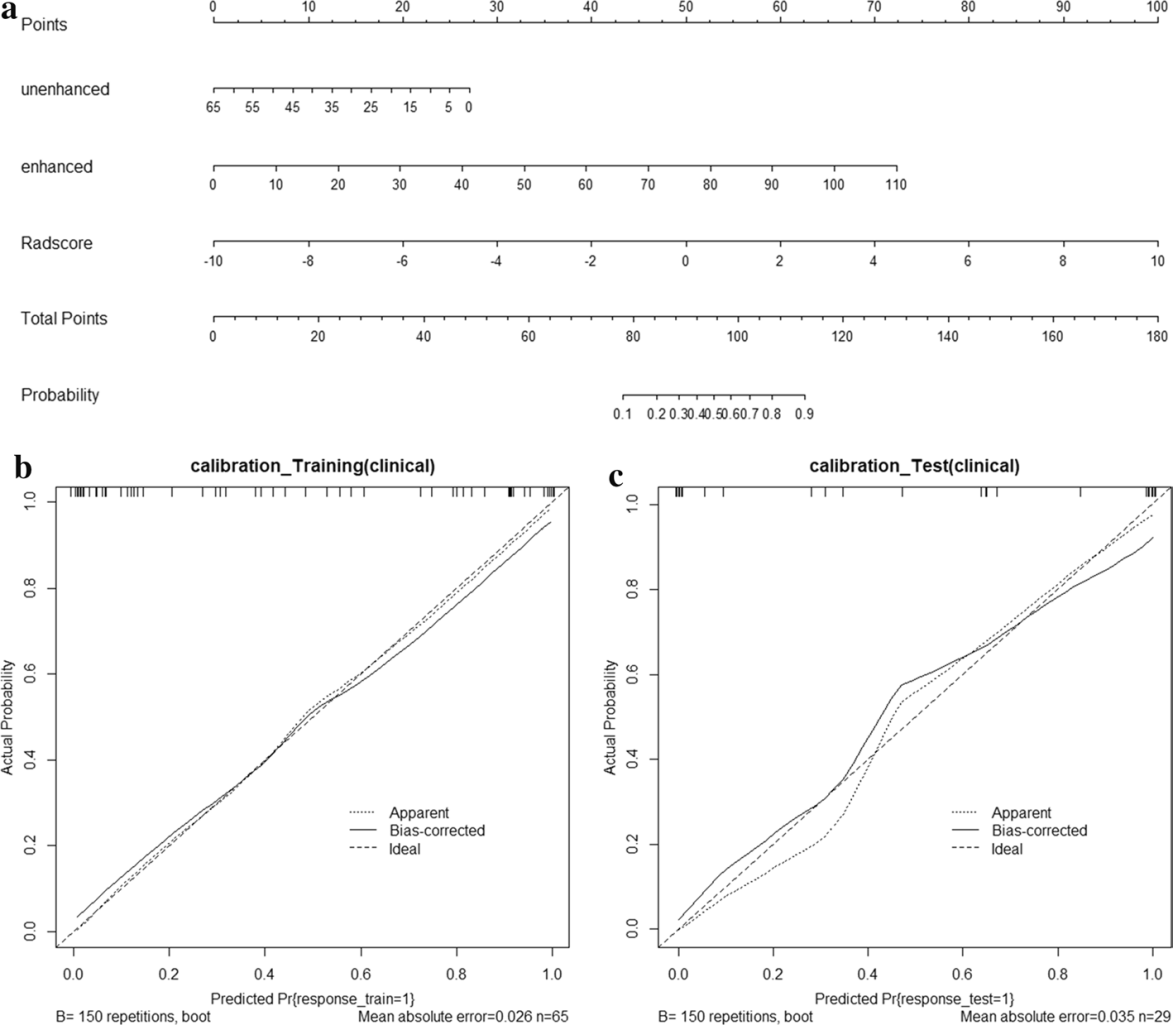
Combined nomogram and calibration curve of the enhanced CT. **a** The developed combined nomogram to predict the probability of the differential diagnosis between the anterior mediastinal cysts and type B1 and B2 thymomas. By summing the scores of each point and locating it on the total score scale, the estimated probability of the differential diagnosis could be determined; **b**, **c** calibration curves to predict the training and test datasets. The 45° straight line represents the perfect match between the actual (*y*-axis) and nomogram-predicted (*x*-axis) differential diagnosis probabilities. A closer distance between two curves indicates higher accuracy prediction and actual observation for the anterior mediastinal cysts and type B1 and B2 thymomas in both the training and test datasets

A combined nomogram of the enhanced CT was constructed based on radscore, unenhanced CT value, and enhanced CT value. The combined nomogram and calibration curves also indicated good agreement between the nomogram prediction and actual observation in both the training and test datasets (Fig. 5). For the training dataset, a non-significant statistic (*P* = 0.735) of the Hosmer–Lemeshow test indicated no significant deviation from an ideal fitting. The AUC value was 0.941 (95% CI 0.853–0.984). For the test dataset, a non-significant statistic (*P* = 0.602) and an AUC of 0.938 (95% CI 0.781–0.993) were obtained (Table 2). Using the bootstrap method, the AUC values were consistent with above results in both training and test dataset (mean ± standard deviation: training dataset: 0.941 ± 0.021; test dataset: 0.938 ± 0.033).

## Discussion

In this study, a nomogram based on enhanced CT was developed and validated using radiomics method for quantified the probability of the differential diagnosis of anterior mediastinal cysts and type B1 and B2 thymomas. The combined nomogram was constructed by incorporating the radscore and the two clinical features of unenhanced CT value and enhanced CT value. The radscore was calculated from the CT images, which was developed by the selective image features. The combined model of enhanced CT images yielded an optimal AUC in both training and test datasets (training dataset: 0.941, test dataset: 0.938). Invasive procedures such as endoscopic biopsy of the mediastinal mass are dangerous, because these masses are near the heart and mediastinal great vessels [22, 23]. Thus, a non-invasive biomarker that can be obtained preoperatively to diagnose anterior mediastinal cysts from type B1 and B2 thymomas will be valuable in clinical practice.

For our study, we found that the extracted features of the unenhanced and enhanced CT images were different, and the models had different efficiency. The AUC of the unenhanced radscore model were 0.823 in training dataset and 0.856 in test dataset, respectively, and 0.928 and 0.899 for enhanced radscore model, which were higher than those of the unenhanced radscore model. The AUCs of the training and test datasets of the unenhanced combined model were 0.933 and 0.928, respectively, and the AUCs of the training and test datasets of the enhanced combined model were 0.941 and 0.938, respectively, also higher than those of the unenhanced model. The results suggested that the features of enhanced phase CT imaging can better reflect the internal heterogeneity of AMC than unenhanced phase CT imaging. The AUCs of the combined model both in the unenhanced and enhanced phases were higher than those of the radscore model, the combined models included not only image features but also unenhanced CT and enhanced CT values of the mediastinal window of clinical features, indicating that the clinical features of the CT value was a very important biomarker for the differential diagnosis between anterior mediastinal cysts and type B1 and B2 thymomas. As the nomogram illustrates, the radscore accounted for most of the proportion compared with the other clinical features, making the radiomics signature as a cardinal biomarker to predict the differential diagnosis of AMC and type B1 and B2 thymomas. The higher the total points of the nomogram are, the more likely type B1 and B2 thymomas are to be diagnosed.

Yasaka et al. [24, 25] studied that the enhanced phase CT could better reflect the internal heterogeneity of anterior mediastinal thymomas and other masses than the unenhanced phase using quantitative computed tomography texture analysis to estimate the histological subtypes of thymic epithelial tumours and differentiation between solid masses and cysts. Only few scholars have conducted relevant research in the radiomics field. Wang et al. [26] obtained the results that the AUCs were 0.829 and 0.860 for the radiomics signature based on unenhanced and enhanced CT images in differentiating advanced stage thymomas from early stage thymomas, respectively. However, Sui et al. [27] believed that the unenhanced phase could better distinguish high-risk and low-risk thymomas than the enhanced phase, because more texture features were selected from the unenhanced phase than from the enhanced phase, and tumour heterogeneity was better detected in the unenhanced phase, the above studies were similar to our results that the enhanced CT and unenhanced CT radiomics can better differentiating the different stages of thymoma, the enrolled thymomas were confirmed by Masaoka clinical stage and WHO histologic classification. In our study, only AMC distinguished from type B1 and B2 thymomas was enrolled to research, because most of type B3 thymic carcinomas can easily be distinguished from cysts preoperatively by CT manifestations. Otherwise, type B1 and B2 thymomas often have a complete capsule even on enhanced CT imaging, making it difficult to distinguish them from AMC. Through this study, the AUCs of combined model distinguishing AMC distinguished from type B1 and B2 thymomas in enhanced and unenhanced CT were all greater than 0.9 in training and test datasets, the sensitivity and specificity were also greater than 0.8. More sample sizes and multi-center external data will be included to further validate our results.

The seven feature parameters selected in this study reflected the distribution of the image grey value, texture features and spatial differences of VOI [28–30]. The feature parameters extracted from the unenhanced phase were the Quantile0.025 and VoxelValueSum of the histogram texture, the feature parameters extracted from the enhanced phase were the RMS, VoxelValueSum of histogram texture and SurfaceVolumeRatio of formfactor texture. The feature parameters extracted from the unenhanced and enhanced phases both included the VoxelValueSum, also indicating that the tumour size had important contributions for differentiating between AMC and thymomas. The coefficient SurfaceVolumeRatio extracted from the enhanced phase was − 0.951 and was negatively correlated with the proportion of thymoma diagnosed, thus indicating a more subglobular mediastinal lesion and a greater likelihood of a thymoma diagnosis, which was consistent with a radiologist’s diagnosis by routine imaging [31]. GLCM is defined by the joint probability density of pixels at different positions, reflecting comprehensive information about the direction and amplitude of the imaging grey distribution, mainly reflecting the influence of pixels on spatial dependence and the relationship with the surrounding environment. The feature parameters extracted from the unenhanced phase were GLCMEntropy_angel0_offset1 and HaralickCorrelation_AllDirection_offset1_SD of GLCM texture, which indicated the heterogeneity of the lesion and degrees of complexity and similarity of the greyscale distribution; this features reflect the degree of the difference of the internal details of the lesion from different aspects, the critical factor to distinguish between AMC and thymomas.

Our study still had several limitations. First, the patients were collected from a single institution retrospectively and the number of patients included in our study was also small, the statistical results reflected in our results may be limited, further larger sample size and multi-center are needed to test the proposed model. Second, we cannot explain the selected feature results to clinicians and patients reasonably.

## Conclusion

In summary, the combined model based on enhanced CT and clinical factors as a non-invasive biomarker may provide a potential tool to facilitate the differential diagnosis of anterior mediastinal cysts and type B1 and B2 thymomas. With further clinical research, a radscore model may provide complementary diagnostic information and help to avoid unnecessary surgical resection for patients with anterior mediastinal cysts.

## Methods

### Subjects

This retrospectively study was approved by the ethics committee of the hospital, and the requirement for informed consent was waived.

188 patients with AMC or type B1 and B2 thymomas confirmed by pathology in the department of thoracic oncology at our hospital from January 2010 to December 2018 were collected. The inclusion criteria were as follows: (1) complete routine unenhanced and enhanced chest CT images; (2) round and uniformly dense lesions without infiltration of surrounding tissues. (3) All included AMC misdiagnosed as thymomas and underwent resection. Patients with incomplete CT images were excluded. Finally, 188 patients were included in the study, 84 males and 104 females, mean aged 52 (19–70) years.

Baseline clinical features were derived from our medical records, including age, sex, primary site (left or right), lesion size, unenhanced CT value, enhanced CT value and change of CT value (enhanced CT value minus unenhanced CT value).

### Examination methods

Unenhanced and 1-phase enhanced chest CTs were performed using a Siemens Definition Flash 64 row. The scanning sequences were the following parameters: tube voltage 120 kV, tube current 250 mAs, 5-mm section collimation, field of view, 300 mm, matrix, 512 × 512, pixel size, 0.68 × 0.68 mm. 38-s delay scan was for enhanced phase CT scan after the administration of 100 to 120 mL of 300 mg/mL iodinated contrast material (Loversol Injection; Liebel-Flarsheim Canada Inc.) at a 3-mL/s injection rate with a pump injector. All patients were scanned with the same machine using identical scanning parameters to ensure the same imaging parameters.

### VOI segmentation and radiomics feature extraction

**Fig. 6:**
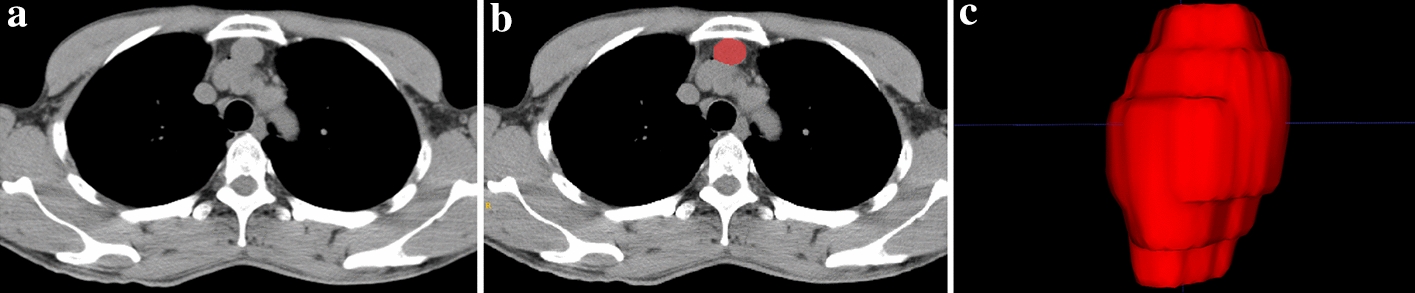
Lesion segmentation. **a** CT images were acquired first; **b** radiologists manually draw a region slice-by-slice that encloses the contour of lesion; **c** lesion segmentation in 3D-VOI

The chest CT images were obtained from the Picture Archiving and Communication Systems (PACS) database. For both the unenhanced phase and enhanced phase CT images of the mediastinal window, a 3D volume of interest (VOI) manual segmentation was performed using ITK-SNAP software (Version3.4.0, http://www.itksnap.org/) (Fig. 6). When multiple tumours were present, the largest diameter tumour was used to analyse.

We randomly chose 60 unenhanced and enhanced CT images for intraclass correlation coefficient (ICC). The segmentation was performed independently by two experienced radiologists. Intra-observer ICC was computed by comparing two extractions of reader A (10 years of experience in chest CT). Inter-observer ICC was computed by comparing reader A and reader B (15 years of experience in chest CT). When the ICC was greater than 0.75, it was considered good agreement, and the remaining 128 image segmentation was performed by reader A. We then obtained two feature sets (feature set 1 of 188 overall patients were extracted by reader A and feature set 2 of 60 randomly images by reader B). The feature set 1 was used to perform the model training and feature set 2 was used to test the robustness and reproducibility of features from set 1.

Image processing was applied before feature extraction, including image resample to 1 × 1 × 1 mm^3^ voxel size and image grey normalization to uniform greyscale of 0–255. A total number of 180 image features were extracted for each patient from the enhanced and unenhanced CT images based on VOI by AK software (Artificial Intelligence Kit V3.0.0.R; GE Healthcare). The feature set included histogram features (number = 42), grey level co-occurrence matrix (GLCM) features (number = 58), grey-level run-length matrix (RLM) features (number = 60), formfactor features (number = 9) and grey-level size-zone matrix (GLZSM) features (number = 11) [32]. These features could characterize intratumour heterogeneity, may contain the underlying genotypes and protein structures [33, 34].

### Feature selection and radiomics signature construction

To eliminate the differences in the value scales of extraction features, feature normalization was performed before feature selection, each feature for all patients was normalized with *Z* scores subtracting the mean value and divided by standard deviation [35].

All the patients were randomly divided into the training (*n* = 130) and test (*n* = 58) datasets at a ratio of 7:3 [36]. The feature selection and radiomics signature construction was performed in the training dataset. Four steps were used to feature selection. First, the ICC was used to select the robustness and reproducibility features to reduce the manual segmentation among different radiologists [37]. ICC greater than 0.75 indicated a high correlation according to the thumb rule [38]. Second, univariate logistic regression was used to select the independent risk features with *P* < 0.05. Third, correlation analysis was conducted on any two features, when the correlation coefficient was greater than 0.9, excluding one of them. The final step method was least absolute shrinkage and selection operator (LASSO) [39] to further select the most useful features by penalty parameter tuning *λ*, we chose the optimal *λ* based on the minimum criteria according to tenfold cross-validation. This method was widely used for the radiomics analysis of high-dimensional features but small medical images.

The selected features were used to construct the radscore model. A radiomics signature (radscore) was then calculated for each patient via a linear combination of selected features that was weighted by their respective coefficients.

### Construction and validation of combined model

Univariate logistic regression was used for seven clinical features in the training datasets, including gender, age, primary site, lesion size, unenhanced CT value, enhanced CT value and change of CT value, to select independent clinical predictors. Multivariable logistic regression analysis combining above independent clinical risk factors and radscore was applied to develop combined model for the differential diagnosis between AMC and type B1 and B2 thymomas [40]. To detect the multi-collinearity between variables in the combined model, the variance inflation factor (VIF) was used to perform the collinearity diagnosis with the VIFs > 10 indicating a severity collinearity [41].

The discrimination and calibration curve were used to evaluate the performances of the clinical models, radscore models and combined models (unenhanced and enhanced CT) in the training and test datasets. The discrimination performance was accessed by receiver operating characteristic (ROC) curve and area under the curve (AUC), accuracy, sensitivity and specificity. To estimate the predict error, we further tested the proposed model of enhanced CT using a 1000-iteration bootstrap analysis in both datasets of enhanced CT. For each repetition, a random subset of 50% patients from training or test was selected and the corresponding AUC was calculated [42]. Furthermore, nomogram of the combined model of enhanced CT was constructed. The calibration curve was used to detect the consistency between the predicted and actual AMC probability, which was quantitatively evaluated by Hosmer–Lemeshow test indicating the goodness of model fit when *P* > 0.05 [43].

### Statistical analysis

All statistical analysis was executed by R software (version 3.0.1; http://www.Rproject.org). Univariate analysis for clinical features was performed using independent sample *t* test or the Mann–Whitney *U* for continues variable and Chi-squared test for categorical variable (sex, primary site). The statistical significance levels were all two-sided, with statistical significance set at 0.05 [44]. Multivariate logistic regression analysis was performed using the “stats” package. Nomogram construction was performed using the “rms” package.

## Data Availability

The datasets generated and/or analyzed during the current study are not publicly available due to an IRB decision which was made in the interest of ensuring patient confidentiality but are available from the corresponding author on reasonable request.

## Abbreviations

AMC: Anterior mediastinal cysts
AUC: Area under the curve
AK: Artificial intelligence kit
CT: Computed tomography
CI: Confidence interval
GLCM: Grey-level co-occurrence matrix
GLZSM: Grey-level size-zone matrix
HU: Hounsfield unit
ICC: Intraclass correlation coefficient
LASSO: Least absolute shrinkage and selection operator
PACS: Picture archiving and communication systems
ROC: Receiver operating characteristic
ROI: Region of interest
RLM: Run-length matrix
SD: Standard deviation
WHO: World Health Organization
3D: Three dimensions
